# Yellow Fever at New Orleans

**Published:** 1853-09

**Authors:** 


*Yellow Fever at New Orleans.*—All our readers are aware of the extensive prevalence and appalling fatality of this epidemic in the city of New Orleans, at the present time. We have no sources of information respecting it except the newspaper intelligence. The next issue of the New Orleans Med. Journal will probably contain a more accurate account than can be gleaned from the daily prints, and we accordingly defer giving any details.

